# Altitudinal Variation on Metabolites, Elements, and Antioxidant Activities of Medicinal Plant *Asarum*

**DOI:** 10.3390/metabo13121193

**Published:** 2023-12-09

**Authors:** Liben Pan, Nan Yang, Yushu Sui, Yi Li, Wen Zhao, Liqiu Zhang, Liqiang Mu, Zhonghua Tang

**Affiliations:** 1 School of Forestry, Northeast Forestry University, Harbin 150040, China; pan7216@nefu.edu.cn (L.P.); 1413085125@nefu.edu.cn (Y.L.); zhaowen186@nefu.edu.cn (W.Z.); 2 Key Laboratory of Forest Plant Ecology, Ministry of Education, Northeast Forestry University, Harbin 150040, China; nanyang@ibcas.ac.cn; 3 College of Chemistry, Chemical Engineering and Resource Utilization, Northeast Forestry University, Harbin 150040, China; suiys@nefu.edu.cn; 4 School of Medicine and Pharmacy, Tonghua Normal University, Tonghua 134002, China; liq920309@gmail.com

**Keywords:** *Asarum*, altitude, metabolism, phyto-chemicals, asarinin, sesamin

## Abstract

*Asarum* (*Asarum sieboldii* Miq. f. *seoulense* (Nakai) C. Y. Cheng et C. S. Yang) is a medicinal plant that contains asarinin and sesamin, which possess extensive medicinal value. The adaptation and distribution of *Asarum*’s plant growth are significantly affected by altitude. Although most studies on *Asarum* have concentrated on its pharmacological activities, little is known about its growth and metabolites with respect to altitude. In this study, the physiology, ionomics, and metabolomics were investigated and conducted on the leaves and roots of *Asarum* along an altitude gradient, and the content of its medicinal components was determined. The results showed that soil pH and temperature both decreased along the altitude, which restricts the growth of *Asarum*. The accumulation of TOC, Cu, Mg, and other mineral elements enhanced the photosynthetic capacity and leaf plasticity of *Asarum* in high-altitude areas. A metabolomics analysis revealed that, at high altitude, nitrogen metabolism in leaves was enhanced, while carbon metabolism in roots was enhanced. Furthermore, the metabolic pathways of some phenolic substances, including syringic acid, vanillic acid, and ferulic acid, were altered to enhance the metabolism of organic acids. The study uncovered the growth and metabolic responses of *Asarum* to varying altitudes, providing a theoretical foundation for the utilization and cultivation of *Asarum*.

## 1. Introduction

Global climate change has had a significant impact on ecological environments, leading to changes in the physiological–ecological adaptations and distribution ranges of plants. Altitude has a regular impact on ecological factors, such as temperature, water, and UV radiation, by affecting the vertical variation of water and heat in the environment [1,2]. Altitude-dependent natural climate gradients serve as space-for-time substitutions, allowing for potential characteristics of plant responses to temporal climate change to be inferred [3,4]. Therefore, examining how plants respond to existing climatic gradients is critical for understanding and predicting whether and how plants may persist in severe climate change.

Plants adapt to environmental change by overcoming environmental stresses through the phenotypic plasticity and variation in physiological and metabolic pathways. For instance, the leaf area and specific leaf area of plants decreased significantly with an increasing altitude gradient [5]. Previous studies have shown that a higher altitude promotes the synthesis of flavonoids, which adjusts the antioxidant capacity of plants [6]. Golob et al. [7] found that, in high-altitude areas, plant investment in secondary metabolites increased, while the investment in primary metabolites decreased.

Medicinal plants are no exception. Several studies have shown that the synthesis and accumulation of active ingredients in medicinal plants are closely related to their specific climatic environment [8,9,10]. Zhao et al. [11] examined the response of the medicinal plant *Herpetospermum pedunculosum* (Ser.) C. B. Clarke to altitude gradients and found that its capacity for adaptation at high elevations is directly correlated with amino acid and carbon metabolism. Kumari et al. [12] detected *Picrorhiza kurroa*’s primary and secondary metabolites and found that source-sink carbon partitioning, the tricarboxylic acid (TCA) cycle, ascorbate metabolism, and other metabolic pathways participate in the adaptation of plants to alpine environment. In addition, essential and trace elements are important active, primary, and secondary components in medicinal plants [13]. A variety of mineral elements have been reported to be closely related to the enhancement of plant stress tolerance. For example, Ca, B, Mn, and Cu are closely related to the flavonoid composition of plants and their content, antioxidant capacity, and cell wall synthesis [14,15,16].

*Asarum* (*Asarum sieboldii* Miq. f. *seoulense* (Nakai) C. Y. Cheng et C. S. Yang) is a perennial herb of the genus *Asarum* of Aristolochiaceae, mainly distributed in northeast China and Korea [17,18]. *Asarum* is widely used clinically as an herbal medicine for the treatment of colds, headaches, toothaches, rheumatism, and coughs, which is attributed to a number of active constituents, including essential oils, acid amides, and lignans (especially asarinin and sesamin) [19]. Asarinin is the main medicinally active ingredient in *Asarum*, which is classified as a quality control standard for Pharmacopoeia [20]. Asarinin and sesamin have been shown to have antiviral, allergic, and therapeutic effects on arthritis [21]. The current research on *Asarum* mainly focuses on the extraction of chemical components and pharmacological activities [22]. However, little is known about the changes in the physiological and medicinal active substances in *Asarum* caused by altitude. 

This study employed physiological, metabolomic, and ionomic methods to investigate the adaptability and survival of *Asarum* roots and leaves under different altitude gradients. The assessment involved measuring the biomass, antioxidant capacity, elemental content, active substances, primary metabolites, and phenolic metabolites to uncover adaptive mechanisms and survival strategies. The results have key implications for future research and the comprehension of *Asarum*’s survival strategies.

## 2. Materials and Methods

### 2.1. Growth Condition and Plant Materials

The study area, Sifang Mountain, was located in the Laoling Mountains of South Changbai Mountain, in the northeast of Tonghua City, Jilin Province. The experimental area belongs to the East Asian monsoon climate, influenced by the topography of Changbai Mountain. The continental climate is obvious: the average annual temperature is 5.5 °C, and the annual precipitation is 880 mm. The soil types are slightly acidic and neutral, and the vegetation types mainly include deciduous broad-leaved forest and mixed coniferous broad-leaved forest. Based on altitudes, the samples were divided into three groups: L (620.79–625.50 m), M (827.60–834.94 m), and H (1020.37–1022.74 m). In this study, based on the longitude and latitude of the sample plots, we collected mean annual temperature (MAT), mean annual precipitation (MAP), and mean annual solar radiation (MASR) under different altitudes at Worldclim (https://www.worldclim.org/, (accessed on 12 September 2023)). Detailed description of sample plots is shown in Table 1.

*Asarum* (*Asarum sieboldii* Miq. f. *seoulense* (Nakai) C. Y. Cheng et C. S. Yang) is a perennial herb capable of both sexual reproduction through the production of seeds, and vegetative reproduction by the rhizomes. *Asarum* was collected at different elevations between 10–12 am in clear weather on 22 May 2022. Five replicate 10 × 10 m plots were demarcated at each site. Expanded and healthy leaves of similar flowering periods and fibrous roots with a diameter of 1–2 mm were collected from each plot. The samples were stored using two methods. One sub-sample was frozen immediately in liquid nitrogen and stored at −80 °C for metabolomic analyses. The second sub-sample was dried in an oven at 60 °C until constant weight and then ground into powder. 

Samples of soil were taken at every altitude. At each sampling site, soil was collected from the 0–20 cm soil layer using a stainless-steel soil corer, providing a total of five soil cores per plots. Soil samples from each plot were mixed to form a total of five composite samples at each elevation, and then removed to check for visible roots, rocks, and litter. Samples of soil were air-dried and put through a 0.25 mm sieve, facilitating comprehensive analysis of the soil’s physico-chemical characteristics.

### 2.2. Determination of Nutrient Element and Soil Properties

The electrical conductivity (EC) and pH value of soil sample were determined using a conductivity meter (DDS-307, Shanghai, China) and a pH meter (PHS-3C, Shanghai, China), respectively. 

The total organic carbon (TOC) and total nitrogen (TN) content in plant and soil samples were determined using an elemental analyzer (Elementar Inc., Hanau, Hessen, Germany). The total phosphorus (TP) content was determined using a UV spectrophotometer after digestion with H_2_SO_4_ and H_2_O_2_ [23]. The determination of other elements (Mg, Al, Fe, Zn, Mn, Cu, B, and Mo) was performed by inductively coupled plasma optical emission spectroscopy (ICP OES Optima 8 × 00) (PerkinElmer, Inc., 10 Waltham, MA, USA) [24]. The plant powder 0.25 g (0.15 g of soil sample), moistened with water, was added with 5 mL of HNO_3_ and 1 mL of HClO_4_ (and 2 mL of HF for soil samples), and then nitrated using a hotplate at 200 °C until the contents became viscous. After cooling, 10 mL of 2% (*v*/*v*) HNO_3_ was added to ensure that the contents were completely dissolved, then transferred to a 100 mL volumetric flask, and ultra-pure water was added to scale for elemental determination. ICP operation method was as follows: RF power 1200 W; plasma Argon (Ar) gas flow rate 12 L/min; auxiliary gas flow rate 1 L/min; nebulizer Ar gas flow 0.7 L/min; and sample uptake rate of 0.8 L/min. Elemental standards with different concentration gradients were established and standard curves were calculated to obtain the concentration of the elements in the samples.

### 2.3. Determination of Growth and Physiological Indices

#### 2.3.1. Growth Indices Measurement

The leaves and roots of *Asarum* were dissected and dried to constant weight at 60 °C, and the biomass of the leaves and roots were weighed. Healthy and complete leaves were selected from each altitude; leaf area was determined using a Uniscan M1 violet scanner, and then were dried and weighed.

#### 2.3.2. Extraction and HPLC Analysis of Asarinin and Sesamin

The root and leaf of *Asarum* samples (0.5 g) were combined with 25 mL of 70% (*v*/*v*) methanol and extracted for 1 h in an ultrasonic bath. After centrifuging the extract for 10 min at 8000 rpm, the resulting solution was filtered through 0.45 µm membranes in preparation for HPLC injection. A Zorbax SB-C18 (5 µm, 4.6 mm × 25 cm, Agilent Technologies, Santa Clara, CA, USA) column was used for the separation. The column temperature was 30°C and the flow rate was maintained at 1.0 mL/min. Methanol (solvent B) and phosphoric acid (solvent A) at a ratio of 0.05% (*v*/*v*) made up the mobile phase. The injection volume was 10 μL, and peaks were observed at 245 nm. The following was the gradient program: 70% A/30% B 3–6 min, and 60% A/40% B 0–3 min. The peaks of the samples were identified based on the matching retention times compared to those of the authentic standards.

#### 2.3.3. Total Phenolic and Total Flavonoid Contents

The dried powder (0.2 g) of the *Asarum* sample was mixed with 10 mL of 70% methanol for 15 min and centrifuged at 8000 rpm for 10 min at 4 °C. After collecting the supernatant, the process was repeated one more time. Total phenol and flavonoid contents were determined from the methanolic extracts. The modified Folin–Ciocalteu method was used to determine the total phenolic content [25]. The reaction mixture contained 5 mL of 10% (*v*/*v*) Folin–Ciocalteu reagent, 4 mL of 7.5% (*v*/*v*) Na_2_CO_3_, 2.5 mL deionized water, and 1 mL of the methanolic extract. The reaction mixture was incubated in the dark for 1 h to develop color and the absorbance was determined at 760 nm. Gallic acid was used to prepare the standard curve. The aluminum chloride reaction was used to calculate the total flavonoid content [26]. To the supernatant (0.5 mL), 0.15 mL of 5% NaNO_2_ solution was added and incubated for 5 min, followed by 0.15 mL of 10% AlCl_3_-6H_2_O solution. After 5 min, 1 mL of 1 M NaOH solution was added to the reaction mixture. The above reactions were carried out at room temperature. The absorbance was measured at 415 nm and a standard curve was constructed using rutin standards.

#### 2.3.4. Antioxidant Activity

The 2,2-diphenyl-1-picrylhydrazyl (DPPH) radical scavenging rate was determined by referring to the method of Szabo et al. [27]. The methanol extract was diluted with 70% (*v*/*v*) methanol to a concentration gradient of 60, 80, 100, 200, and 300 μg/mL, and three replicates were set up for each group. As a positive control, 10–100 μg/mL of Trolox was obtained from Sigma-Aldrich Chemical Co. (St. Louis, MO, USA). For each group, 1 mL was mixed with 3 mL of distilled water and 6 mL of 0.1 mM DPPH solution, and the absorbance values were measured in a water bath at 37 °C for 30 min, and then we measured the absorbance at a wavelength of 517 nm. The Dong et al. method [28] was used to determine the 2,2′-azinobis (3-ethylbenzothiazoline-6-sulfonic acid) (ABTS) radical scavenging rate. The methanol extract was diluted with 70% methanol to a concentration gradient of 60, 100, 200, 300, 400, and 600 μg/mL, and three replicates were set up for each group. Trolox, at a concentration of 5–200 μg/mL, serves as a positive control. Then, 2 mL of each extract was mixed with 2 mL of ABTS, and the reaction was performed at 25 °C for 6 min with protection from light, and we measured the absorbance value at a wavelength of 734 nm. A fitted curve function was constructed to calculate the effective concentration required when the clearance of DPPH and ABTS was 50% (50% inhibiting concentration, IC50).

### 2.4. Metabolite Extraction and Detection

#### 2.4.1. Primary Metabolite Analysis

In 0.1 g of sample was added 0.8 mL of 80% (*v*/*v*) methanol solution and 5 μL of 2-chloro-L-phenylalanine (0.3 mg/mL), sonicated at 4 °C for 30 min, then centrifuged for 15 min at 4 °C and 12,000 rpm. The 200 μL supernatant was taken in a 1.5 mL centrifuge tube, concentrated by rotary evaporation; 35 μL of 20 mg/mL methoxypyridine solution was added, and the reaction was allowed to proceed for 90 min at 37 °C with vigorous shaking for 30 s. Then, 35 μL of N, O-Bis (trimethylsilyl) trifluoroacetamide (BSTFA) (containing 1% TMCS) was added for derivatization and the reaction was carried out at 70 °C for 60 min. The obtained material was then left at room temperature for 30 min, and then analyzed by GC-MS. The GC–MS data were obtained using the Agilent 7890A-5975C (Agilent, USA) and a non-polar DB-5 capillary column (30 m × 250 um I.D., J&W Scientific, Folsom, CA, USA). Instrument parameters are as follows: the temperature is 280 °C, the EI ion source is 230 °C, the carrier gas is high purity helium (purity ≤ 99.999%), the shunt ratio is 10:1, the injection volume is 1.0 μL, and the solvent delay is 5 min. The ramp-up procedure is as follows: initial temperature 70 °C, ramp-up to 200 °C at 10 °C/min, then ramp-up to 280 °C at 5 °C/min, and maintain for 10 min. Solvent delay 5 min. ramp-up procedure is as follows: initial temperature 70 °C, ramp-up to 200 °C at 10 °C/min, then ramp-up to 280 °C at 5 °C/min, and maintain for 10 min.

#### 2.4.2. Phenol Metabolites Analysis

Dried powder (1.0 g) was added to 6 mL of 70% methanol, and the mixture was sonicated and ultra-sonicated for 30 min. The mixture was then centrifuged, with the supernatant being kept. This extract process was repeated twice, and the supernatants were combined. The supernatant from both times was concentrated by rotary evaporation. Subsequently, the dried sample was re-dissolved in 1 mL of 70% methanol. Solution collected was analyzed by UPLC/Q-TOF-MS, with a reversed Acquity UPLC BEH C18 Column (1.7 μm, 2.1 mm × 50 mm), and a Waters ^®^ Xevo G2 QTOF mass spectrometer.

The settings of the UPLC system were 0.1% formic acid (*v*/*v*) in acetonitrile (B) and 0.1% formic acid (*v*/*v*) in aqueous solution (A), with a flow rate of 0.3 mL/min. The employed gradient flow rates were as follows: 0–10 min at 95% A-5% A, 5% B-95% B; 10–18 min at 5% A-95% A, 95% B-5% B; and 18–25 min at 95% A-95% A, 5% B-5% B, respectively. The column temperature was kept at 30 °C, and 2 μL injection volume was used. The conditions of the mass spectrometer were as follows: sampling cone voltage (45 eV), source temperature (400 °C), capillary voltage in positive ESI (+3000 V), and desolvation temperature (500 °C). The scanning range was set between 50–1000 *m*/*z*, with an ion acquisition rate of 0.2 per second. The identification of major compounds was facilitated by their MS/MS spectra and by comparison with authentic standards. 

### 2.5. Statistical Analysis

The presentation of all the data was mean ± standard deviation (SD). One-way ANOVA was used to analyze the data using SPSS 19.0 (SPSS, Santa Clara, CA, USA). In all cases, differences were considered to be significant at a probability level of *p* < 0.05. Plots were made with GraphPad Prism 8. 

The metabolites detected by GC-MS were identified through structural comparison, specifically by comparing the retention time and mass spectra with those of known compounds in the National Institute of Standards and Technology (NIST) library. The LC–MS data were analyzed and normalized through the mass spectrometry software, MassLynxTM 4.1 (Waters Corporation, Milford, DE, USA). This detected peaks and listed both the detected and matched peaks with the retention time and *m*/*z* pair, as well as their intensities.

Multivariate statistics was performed using the Soft Independent Modeling of Class Analogy (SIMCA)-P (version 11.0, Umetrics AB, Umea, Sweden). Prior to principal component analysis (PCA) and partial least-squares discriminant analysis (PLS-DA), all variables were unit variance (UV)-scaled. Differential metabolites were identified based on a threshold variable importance in projection value (VIP > 1.0) and *p*-value (*p* < 0.05). To determine pathway connections, annotated metabolites were mapped to the Kyoto Encyclopedia of Genes and Genomes (KEGG) pathway database (http://www.kegg.jp/kegg/pathway.html, (accessed on 9 September 2023)). 

After log transformation for primary metabolites, phenolic metabolites, two active ingredients, and elements, a two-way ANOVA was performed using SPSS 19.0 to identify analytes for further analysis. The correlation coefficients between analytes were calculated using Pearson’ s correlation analysis. Based on Pearson’ s correlation coefficient (r^2^ > 0.50 and *p* < 0.05), two nodes were considered linked. These relationships were visualized globally for all analytes using Arena3D (http://arena3d.org (accessed on 15 October 2023)) [29].

## 3. Results

### 3.1. Soil Chemical Properties

Along the altitudes, the soil pH was slightly acidic. The total organic carbon (TOC), ranging from 6.84–11.24%, increased with altitude and was demonstrated to be negatively correlated with the pH. An analysis of variance showed that other soil characteristics were significantly affected by the elevation (Table 2). The highest contents of most nutrients, including TOC, total nitrogen (TN), aluminum (Al), iron (Fe), zinc (Zn), manganese (Mn), copper (Cu), and molybdenum (Mo), were observed at high elevations. Notably, TOC, Al, Zn, Cu, and Mo increased with elevation. 

### 3.2. Nutritional Element Distribution and Physiological Indices in Asarum at Different Altitudes

**Figure 1:**
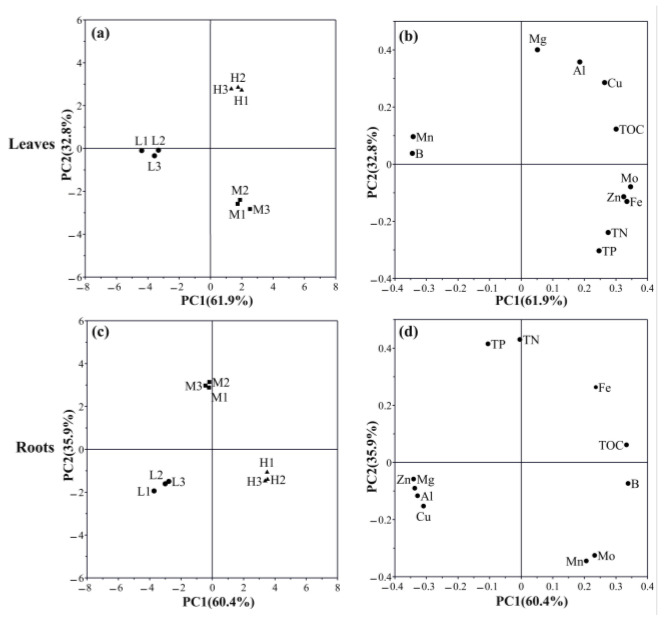
Tissue nutritional element variation analysis using PCA at different altitudes. (**a**) The score plot of elements in leaves; (**b**) the loadings of elements to the PC1 and PC2 in leaves; (**c**) the score plot of elements in roots; and (**d**) the loadings of elements to the PC1 and PC2 in roots.

The principal component analysis (PCA) showed a separation in nutritional element samples at varying altitudes (Figure 1). The leaf and root samples at distinct altitudes were discernibly separated by PC1, which accounted for 61.90% and 60.4% of the total variation (Figure 1a,c). The principal elements contributing to PC1 in leaves were Fe, Mn, Zn, Mo, and B, while, in roots, it was TOC, Mg, Al, Zn, and B (Table S1, Figure 1b,d), respectively. PC2 clearly distinguished the samples of different altitudes in leaves and roots, representing 32.80% and 35.90% of the variations, respectively (Figure 1a,c). The contribution of PC2 was dominated by Mg, Al, and Cu in the leaves, while it was dominated by TN, TP, Mn, and Mo in the roots (Table S1, Figure 1b,d), respectively. 

In addition, the nutritional elements in the leaves and roots of *Asarum* were compared at different altitudes (Table S2). The variation trend of TOC, TN, TP, and Fe along the altitude were consistent in the leaves and roots. The TN, TP and Fe significantly increased at middle altitude, while the highest accumulation of TOC was observed at high altitude. Additionally, the change of Al, Cu, Mg, and B along the altitude were the opposite in the leaves and roots. The content of Al, Cu, and Mg along the altitude significantly increased in the leaves, while it significantly decreased in the roots. 

**Figure 2:**
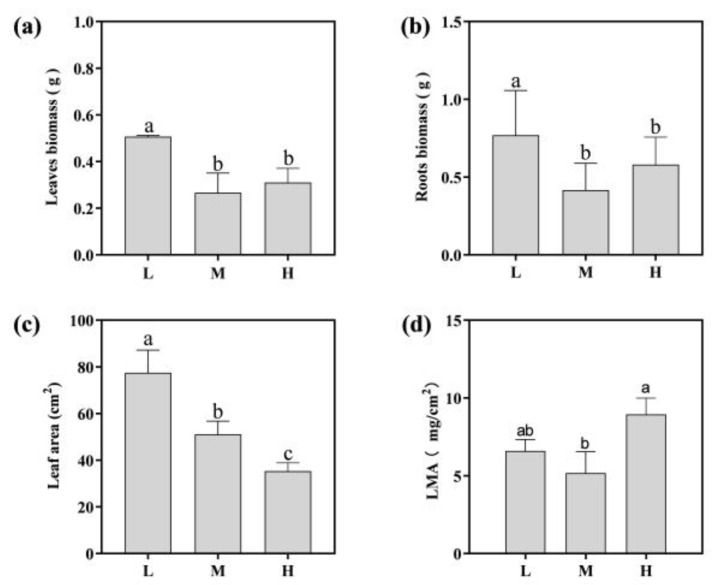
Effect of altitude on leaf biomass allocation (**a**); root biomass allocation (**b**); leaf area (**c**); and specific leaf weight (**d**) in *Asarum*. Different lower-case letters indicate a significant difference (*p* < 0.05) as determined by one-way ANOVA. L indicates low-altitude areas, M indicates middle-altitude areas, and H indicates high-altitude areas.

The growth indices of *Asarum* were also detected (Figure 2). The biomass of the leaves and roots significantly reduced at middle altitude and high altitude (Figure 2a,b). The leaf area decreased by 54.44% at high altitudes compared to low altitudes (Figure 2c). The highest specific leaf weight (LMA) at high altitude was observed (Figure 2d). These results suggest that *Asarum* thrived at low altitudes, whereas higher altitudes had a detrimental effect on its growth.

**Figure 3:**
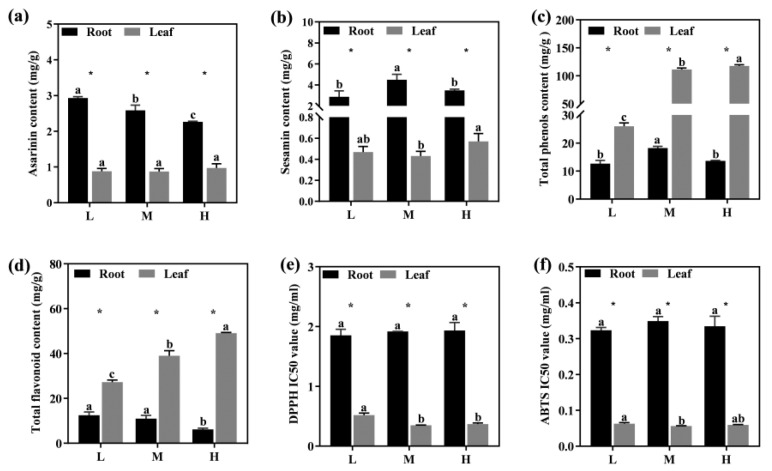
The effect of altitude on the content of asarinin (**a**), sesamin (**b**), total phenols (**c**), total flavonoids (**d**), DPPH IC50 value (**e**), and ABTS IC50 value (**f**) in *Asarum*. Different lower-case letters indicate a significant difference (*p* < 0.05) as determined by one-way ANOVA; * indicates significant differences (*p* < 0.05) between roots and leaves at the same altitude. L indicates low-altitude areas, M indicates middle-altitude areas, and H indicates high-altitude areas.

The medicinal ingredients and antioxidant capacity variables of *Asarum* exhibited varied sensitivity to altitude. The roots were found to contain high levels of asarinin and sesamin, with asarinin being highest at low elevations and sesamin at middle elevations (Figure 3a,b). The contents of total phenols and total flavonoids in the leaves showed a consistent trend with altitude, both significantly increasing with altitude and reaching the highest levels of 117.50 mg/g and 49.70 mg/g at high altitude, respectively (Figure 3c,d). The change trend of the total flavonoid content in roots was the opposite to that in the leaves. (Figure 3d). The antioxidant capacity was evaluated by the IC50 values of DPPH and ABTS. The lower the IC50 value, the stronger the antioxidant capacity. The IC50 of DPPH decreased by 32.54% and 28.41% in middle- and high-altitude leaves (Figure 3e), respectively. The IC50 of ABTS in the leaves decreased by 10.43% and 5.33% in middle- and high-altitude leaves (Figure 3f), respectively, indicating an increase in antioxidant capacity in middle-altitude leaves.

### 3.3. Metabolic Profile in Asarum at Different Altitudes

**Figure 4:**
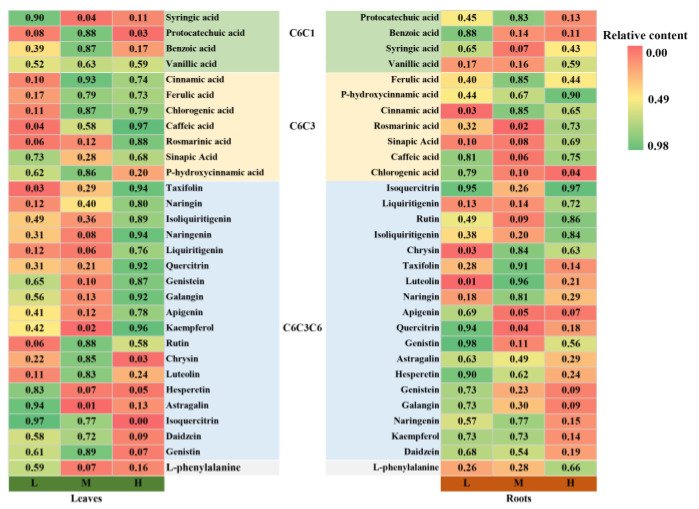
Relative content of phenolic metabolites in leaves and roots at different altitudes. L indicates low-altitude areas, M indicates middle-altitude areas, and H indicates high-altitude areas.

The metabolite profiling of the different altitude samples was conducted using GC-MS and UPLC/Q-TOF-MS. A total of 96 metabolites were identified in the leaves and roots by GC-MS (Table S3), and 30 phenolic metabolites were identified by UPLC-MS-MS (Figure 4). According to the PCA score plots, there was evident variation in the leaves and roots at different altitudes (Figure S1). 

**Figure 5:**
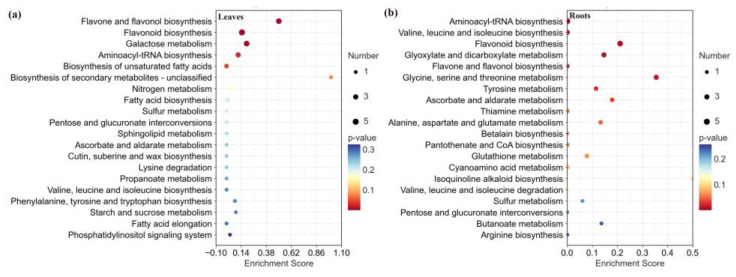
Enriched metabolic pathways of the differential metabolites at different altitudes for leaves (**a**) and roots (**b**). Each bubble in the plot represents a metabolic pathway whose abscissa and bubble size jointly indicate the magnitude of the impact factors of the pathway. A larger bubble size indicates a larger impact factor.

To further screen the differential metabolites, the variable importance in the projection (VIP) by an orthogonal partial least-squares discriminant analysis (OPLS-DA) and the *p*-value by a one-way ANOVA, and metabolites satisfying both VIP > 1 and *p* < 0.05 were considered as differential metabolites. A total of 46 and 45 differential metabolites were identified in the leaves and roots, respectively (Table S4). Moreover, the pathway enrichment analysis of these substances was performed using the Arabidopsis KEGG pathway library (Figure 5). The enrichment pathway in different tissues was significantly different. According to the KEGG annotation and enrichment results, differential metabolites, which might relate to the altitude changes in leaves, were mainly annotated and enriched in flavone and flavonol biosynthesis, galactose metabolism, flavonoid biosynthesis, and aminoacyl-tRNA biosynthesis (−log p > 1, impact > 0.1) (Figure 5a, Table S5). In the roots, there were six biological pathways involved (−log p > 1, impact > 0.1), including flavonoid biosynthesis, ascorbate and aldarate metabolism, glyoxylate and dicarboxylate metabolism, and three amino acid metabolism pathways (Figure 5b, Table S5). 

**Figure 6:**
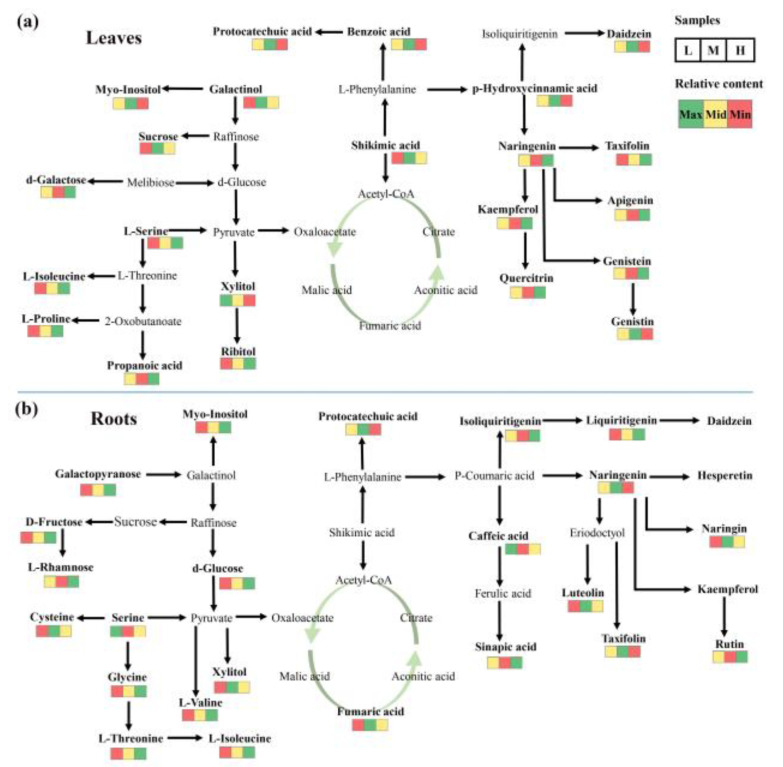
Mapping of differential metabolites on metabolic pathway for leaves (**a**) and roots (**b**). L indicates low-altitude areas, M indicates middle-altitude areas, and H indicates high-altitude areas.

To comprehend the metabolic regulation changes at varying altitudes, we constructed a simple metabolic network diagram (Figure 6). In the leaves, L-serine, L-isoleucine, and L-proline related to nitrogen metabolism accumulated at high altitude, and all of them were involved in the aminoacyl-tRNA biosynthesis pathway, while galactinol and sucrose in the galactose metabolism pathway accumulated more at middle altitude (Figure 6a). In roots, sugars including galactopyranose, D-fructose, L-rhamnose, and d-glucose associated with carbon metabolism accumulated significantly at high altitude (Figure 6b). Significant metabolic responses were found in the phenolic compounds mediated by shikimic acid. With increasing altitude, most of the C6C1 carbon-skeleton phenolic compounds in the leaves tended to decrease, while the C6C3 carbon-skeleton phenolic compounds accumulated (Figure 4 and Figure 6a). Most of the C6C1 carbon-skeleton phenolic compounds and C6C3 carbon-skeleton compounds in the roots showed a similar trend with altitude as in the leaves. The C6C3C6 compounds showed significant tissue specificity in the metabolic response to different altitudes. Most of the them accumulated in the leaves and decreased in the roots at high altitude, such as quercitrin, genistein, galangin, apigenin, and kaempferol (Figure 4 and Figure 6).

### 3.4. Correlations of Metabolites–Elements–Medicinal Ingredients in Asarum

**Figure 7:**
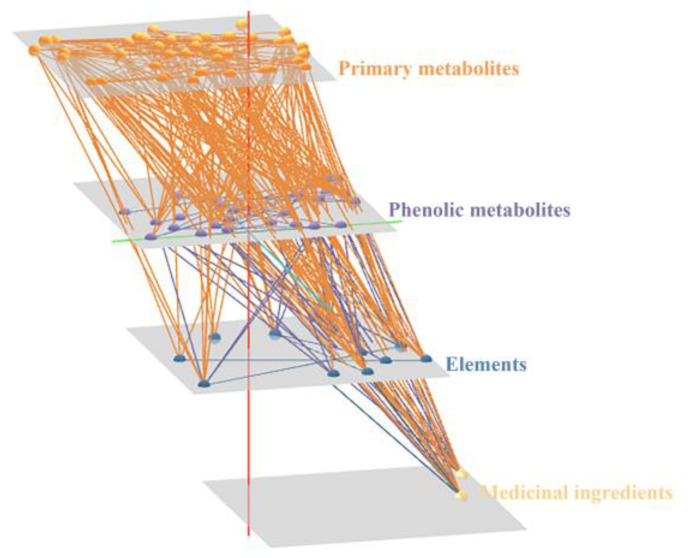
*Asarum* correlation network based on Pearson’s correlation analyses for 83 analytes and 11 elements. Significant correlations are ones with r^2^ > 0.50 and *p* < 0.05. Metabolites and elements are represented by circles, and edges refer to correlations in between. The four layers corresponded to the following compound types: primary metabolites, phenol metabolites, elements, and medicinal ingredients.

To obtain a global overview of the inter-analyte associations, a two-way ANOVA was performed after the log transformation of all metabolites shared in the roots and leaves, selecting analytes that showed significant differences (*p* < 0.05) related to the tissues, altitudes, or altitudes–tissues interactions. A layered network for visualization in three dimensions was constructed based on Pearson’s correlation coefficients (r^2^ > 0.50 and *p* < 0.05), highlighting the connections between composite types (Figure 7). The global network revealed many connections between primary metabolites and phenolic metabolites, as well as between primary metabolites and elements. In addition, node scores were calculated for the nodes in the network, listing the top ten nodes in the MCC ranking (Figure S2). Notably, apart from two medicinal components, organic acids dominated the network.

## 4. Discussion

The effect of altitude on plant growth and the accumulation of metabolic substances are the result of the comprehensive effect of relevant climatic factors (temperature, light, water, etc.) [30]. In this study, there were significant changes in soil physico-chemical properties between different altitudes, with significant alterations in the annual average temperature (MAT) and annual rainfall (MAP) along the altitude gradient. In high-altitude areas, the soil pH was found to decrease, whereas the concentration of soil ions (Al, Fe, Zn, Mn, Cu, and Mo), total organic carbon (TOC), and total nitrogen (TN) substantially increased. This accumulation of TOC may be attributed to the combination of low temperatures and heightened soil acidity at high altitudes, which decelerate the process of the decomposition and mineralization of TOC [31].

The accumulation and distribution of biomass in plants are significantly influenced by external factors [32]. Consequently, comprehending the patterns of biomass accumulation and distribution among plants at diverse altitudes serves as a critical approach to comprehending plant responses to climate alteration. In our study, the underground biomass, aboveground biomass, and leaf area of *Asarum* showed a downward trend along the altitude (Figure 2). A smaller leaf area reduces the solar energy uptake and transpiration rates, thereby reducing the damage to the leaves from high-intensity UV radiation and strong winds [33]. Ullah et al. also obtained similar results in the study of *Primula nivalis*. There exists a substantial negative correlation between morphological features and the altitude, temperature, and sunshine duration, which are the predominant factors influencing plant morphological attributes and resource allocation [34]. Moreover, we also observed the highest specific leaf weight (LMA) at the high altitude (Figure 2d). The LMA can reflect the balance between plant growth and nutrient absorption [35]. In high-altitude areas, a high LMA is usually associated with enhanced defense. Plants will reduce growth and invest more resources in enhancing defense to help them survive in areas with strong radiation and low temperature [36].

The relationship between plant size in high-altitude regions and nutrition has been demonstrated [31]. Plant growth is restrained at high altitudes, which may be due to the harsh climatic conditions and low temperatures at high altitudes, diminished microbial activity, and diminished plant nutrient availability [37]. TOC and Mo in the roots and leaves were significantly accumulated at the highest altitude, while Al, Cu, and Mg decreased in the roots and increased in the leaves at high altitude. Leaf carbon is a representative of leaf toughness and structural compound investment [38], indicating higher plasticity and stress resistance in *Asarum* at high-altitude areas. Mo has been shown to regulate the antioxidant mechanisms and osmotic balance in plants and increase the chlorophyll and carotenoid content, as well as the photosynthetic rate [39]. Meanwhile, Cu and Mg are also transferred to the leaves in the roots at high altitude. Cu and Mg are essential nutrients for plants and participate in photosynthesis, particularly in chlorophyll synthesis and photosynthetic electron transfer [40]. Therefore, the accumulation of the elements at high altitude contributes to the enhancement of photosynthetic capacity and leaf defense. In addition, we observed that the content of B is generally higher in soils with different altitude gradients, and a significant accumulation of B in the roots increases with altitude. B is related to enzyme synthesis and mainly affects physiological processes such as cell wall structure, carbohydrate metabolism, and sugar transport in plants [41]. However, high concentrations of B can cause toxicity in plants and inhibit plant growth, which may be one of the reasons for the inhibition of plant growth in high-altitude areas [42]. Meanwhile, the interactions of B with other elements and metabolites in plants are complex and need to be further investigated.

Plants are always faced with a trade-off between growth and defense, especially in primary and secondary metabolism. Primary metabolites are directly involved in plant growth and development. In this study, galactopyranose, D-fructose, L-rhamnose, and D-glucose were observed to accumulate significantly in the roots at high altitude, suggesting that carbon metabolism is enhanced in the roots of *Asarum* with increasing altitude. Sugars have been shown to be a positive regulatory factor for enhancing plant adaptability [43]. Sucrose is the main product of photosynthesis, providing energy for cell metabolism. Ma et al. demonstrated enhanced photosynthetic rates and sugar accumulation along an increasing elevation gradient [44]. This is consistent with our results. Although sugars primarily function to boost plant adaptability, they operate via distinct regulatory systems. For example, glucose can serve as a respiratory substrate or osmotic agent to maintain cellular homeostasis [45]. Fructose and rhamnose act by forming substrates of secondary metabolites [46].

Similarly, significant alterations in amino acid metabolism subsequent to sugar metabolism were observed in the roots and leaves. In the leaves, L-serine, L-isoleucine, and L-proline, associated with nitrogen metabolism, accumulate at high altitude, and all of them are involved in the biosynthetic pathway of aminoacyl tRNA. Proline, a multifunctional amino acid, functions as a signaling molecule, regulating intracellular osmotic pressure, mitigating protein denaturation, preserving membrane integrity, shielding cells from stress, and curtailing ROS-induced damage [47]. Aminoacyl-tRNA can catalyze the synthesis of amino acids into proteins [48]. Previous studies have shown that proteins can resist low-temperature stress by controlling and activating metabolic pathways related to resistance [4]. Similar results were observed in roots and most of the amino acids including L-threonine, L-isoleucine, and L-valine also accumulated at high altitude. Amino acids and their derivatives serve to activate plant defense mechanisms during plant growth and the development and stress response [49]. Some investigations have suggested that the increase in amino acid levels caused by stress is related to increased nitrogen storage and serves as a precursor for the production of secondary metabolites in plants [50,51,52].

Changes in altitude will alter the accumulation of secondary metabolites in plant tissues, thereby adjusting the adaptability of plants to environmental changes [53]. The increase of reactive oxygen species in high-altitude areas can damage the cell membrane system, resulting in abnormal metabolism, and even cell death [36,54]. The antioxidant activity of medicinal plants is commonly evaluated by the scavenging rate of DPPH radicals and ABTS radicals [55]. In this study, a higher antioxidant capacity was observed in leaves at medium and high altitudes, corroborating the findings of Joshi et al. [56]. Previous studies have shown that phenols and many flavonoids exert antioxidant effects by reducing the accumulation of reactive oxygen species [36,54]. Specifically, our results show that vanillic acid, cinnamic acid, ferulic acid, caffeic acid, rosmarinic acid, sinapic acid, isoliquiritigenin, and rutin accumulated significantly in both the roots and leaves at high altitude. It has been found that cinnamic acid can disrupt the coupling of oxidative phosphorylation to the respiratory chain, which, in turn, affects plant respiration [57]. In our study, the negative impact of high altitude on respiration can be attributed to the interruption of electron transfer chains caused by lower environmental temperatures and high ion accumulation. Ferulic acid is an important insoluble phenolic compound that contributes to the antioxidant activity of plants [58]. Furthermore, ferulic acid may be esterified by cell wall polysaccharides, establishing a link between lignin and cell wall polysaccharides, thereby increasing cell wall rigidity and facilitating the maintenance of expansion pressure [59]. In addition, the accumulation of vanillic acid, ferulic acid, and caffeic acid showed a positive response with increasing altitude in several studies [60,61].

The correlation between mineral elements and metabolite patterns is novel. Previous studies have shown that a variety of elements, including K, Ca, Fe, and Cu, involve many metabolic pathways such as signal transduction, photosynthesis, and carbon assimilation [62,63]. In this study, TN, Al, Cu, Mn, and Mg were significantly correlated with multiple metabolites. Among them, Al, Cu, and Mg were positively correlated with metabolites involved in flavonoid biosynthesis, such as chlorogenic acid, kaempferol, taxifolin, and galangin. Conversely, Al, Cu, and Mg were negatively correlated with compounds involved in glycoxylate and dicarboxylate metabolism, such as oxalic acid, D-glycerate, and succinic acid. In contrast, TN and Mn were positively correlated with compounds involved in glyoxylate and dicarboxylate metabolism. Glyoxylate and dicarboxylate metabolism are involved in the carbohydrate biosynthesis of double fatty acids [64]. Carbohydrates serve as metabolic sources of energy and signaling molecules in plant growth regulation, and are integral to the regulation of plant stress responses [65]. Flavonoid metabolism contributes to the enhancement of antioxidant activity in plants to alleviate oxidative stress [66]. In addition, organic acids such as protocatechuic acid, 2-Butenoic acid, syringic acid, galactaric acid, lactic acid, vanillic acid, and ferulic acid are the key metabolites in the related network. Among them, protocatechuic acid, syringic acid, vanillic acid, and ferulic acid are synthesized through a series of enzymatic reactions through the shikimic acid pathway, with various medicinal values and important sources of antioxidants [67]. Thus, the enrichment of these metabolic pathways could reveal the activation of the corresponding processes or functions, including carbohydrate formation and antioxidant synthesis, which could help to alleviate the inhibition of *Asarum* growth by altitude.

## 5. Conclusions

In summary, different altitudes have a significant impact on the growth and metabolism of *Asarum*. The growth of *Asarum* was inhibited at high altitudes, while the content of total phenols and flavonoids significantly increased with increasing altitude, enhancing its antioxidant capacity. Ionic omics results indicated that, at high altitudes, TOC, Mo, Al, Cu, and Mg in *Asarum* significantly accumulate in the leaves, enhancing their photosynthetic capacity and stress resistance. Based on the results of metabolomics, we found that, in high-altitude areas, the roots mainly activate defense mechanisms by accumulating sugars, amino acids, and organic acids, while sugars in the leaves are reduced and nitrogen metabolism is enhanced. Organic acids play a dominant role in regulating the adaptation strategy of *Asarum* with altitude. Meanwhile, the main active components of *Asarum*, asarinin and sesamin, are mainly accumulated in the roots, with higher levels in low- and medium-altitude areas. These results provide a theoretical basis for explaining the physiological and metabolic response mechanisms of *Asarum* to altitude, and are important for the collection of excellent wild *Asarum* seed sources and their biological research. Nonetheless, the environmental factors at different altitudes are multifaceted, and the response mechanism of *Asarum* to varying environmental factors necessitates further investigation.

## Figures and Tables

**Table 1:** Descriptions of the sampling sites.

Site	Vegetation Type	Elevation (m)	Longitude and Latitude	Aspect	MAT (°C)	MAP (mm)	MASR (kJ m^−2^ day^−1^)	Dominant Species
1	Deciduous broad-leaved forest	620.79– 625.50	126°6′37.67″ N 41°53′54.10″ E	East	3.58	894.00	6907.00	*Erythronium japonicum* Decne., *Polygonatum acuminatifolium* Kom., *Hylomecon japonica* (Thunb.) Prantl & Kündig, *Cardamine leucantha* (Tausch) O. E. Schulz, *Meehania urticifolia* (Miq.) Makino
2	Deciduous broad-leaved forest	827.60– 834.94	126°5′25.39″ N 41°55′50.56″ E	West	2.80	911.00	6941.00	*Erythronium japonicum* Decne., *Anemone amurensis* (Korsh.) Kom., *Anemone raddeana* Regel, *Hylomecon japonica* (Thunb.) Prantl & Kündig, *Lilium distichum* Nakai ex Kamibayashi
3	Deciduous broad-leaved forest	1020.37– 1022.74	126°4′39.67″ N 41°56′1.46″ E	Southeast	2.97	897.00	7263.00	*Gymnospermium microrrhynchum* (S. Moore) Takht., *Erythronium japonicum* Decne., *Maianthemum japonicum* (A. Gray) LaFrankie, *Anemone amurensis* (Korsh.) Kom., *Anemone raddeana* Regel

Note: MAT is mean annual temperature. MAP is mean annual precipitation. MASR is mean annual solar radiation.

**Table 2:** Soil chemical properties at different altitudes.

	LOD (mg/L)	L (Low Altitude)	M (Middle Altitude)	H (High Altitude)
pH	-	6.19 ± 0.18 ^a^	5.77 ± 0.16 ^a^	5.62 ± 0.46 ^a^
EC (ms/cm)	-	1.76 ± 72.99 ^a^	1.32 ± 17.58 ^a^	1.63 ± 11.02 ^a^
TOC (%)	-	6.84 ± 4.53 ^c^	8.88 ± 5.14 ^b^	11.24 ± 1.55 ^a^
TN (%)	-	0.93 ± 0.04 ^ab^	0.81 ± 0.70 ^b^	1.16 ± 0.18 ^a^
TP (%)	-	0.08 ± 0.05 ^b^	0.1 ± 0.03 ^a^	0.06 ± 0.02 ^c^
Mg (mg/kg)	0.0001	4104.79 ± 148.61 ^b^	4948.05 ± 79.02 ^a^	4151.74 ± 62.78 ^b^
Al (mg/kg)	0.0001	33,945.73 ± 1902.79 ^c^	39,451.17 ± 863.20 ^b^	53,979.69 ± 2747.76 ^a^
Fe (mg/kg)	0.0005	20,733.57 ± 1464.33 ^b^	18,692.84 ± 182.27 ^c^	25,240 ± 519.97 ^a^
Zn (mg/kg)	0.0004	78.59 ± 4.03 ^b^	82.71 ± 3.56 ^b^	123.56 ± 11.24 ^a^
Mn (mg/kg)	0.0002	552.36 ± 53.24 ^b^	534.11 ± 9.02 ^b^	850.42 ± 29.29 ^a^
Cu (mg/kg)	0.001	11.22 ± 0.84 ^c^	13.09 ± 0.46 ^b^	16.4 ± 0.76 ^a^
B (mg/kg)	0.0014	35.05 ± 1.08 ^a^	27.27 ± 0.35 ^b^	27.6 ± 2.35 ^b^
Mo (mg/kg)	0.0001	0.53 ± 0.06 ^c^	0.63 ± 0.01 ^b^	0.79 ± 0.06 ^a^

Different lower-case letters indicate a significant difference (*p* < 0.05) as determined by one-way ANOVA. All data shown were the means ± SD. TOC, total organic carbon; TN, total nitrogen; and TP, total phosphorus.

## Data Availability

The data presented in this study are available on request from the corresponding author. The data are not publicly available due to privacy.

## Supplementary Materials

The following supporting information can be downloaded at: https://www.mdpi.com/article/10.3390/metabo13121193/s1, Figure S1: Multivariate analysis of primary metabolites (a,c) and phenolic metabolites (b,d) at different altitudes for leaves and roots; Figure S2: Top 10 nodes in *Asarum* ranked by MCC; Table S1: Variance contribution of each element; Table S2: Nutritional element distribution in leaves and roots at different altitudes; Table S3: List of the metabolites identified in *Asarum*; Table S4: List of the differential metabolites identified in *Asarum*; Table S5: Enriched metabolic pathways of the differential metabolites at different altitudes; Table S6: Statistics for two-way ANOVA, incorporating tissues, altitudes, and their interaction as fixed effects.
