# Supplementary material for: Altitudinal Variation on Metabolites, Elements, and Antioxidant Activities of Medicinal Plant Asarum
Source: Metabolites. 2023 Dec 9;13(12):1193. doi: 10.3390/metabo13121193 (PMC10745449; doi:10.3390/metabo13121193)
Supplement: Supplementary file 1 [file metabolites-13-01193-s001.zip › Supplementary Material A.pdf]

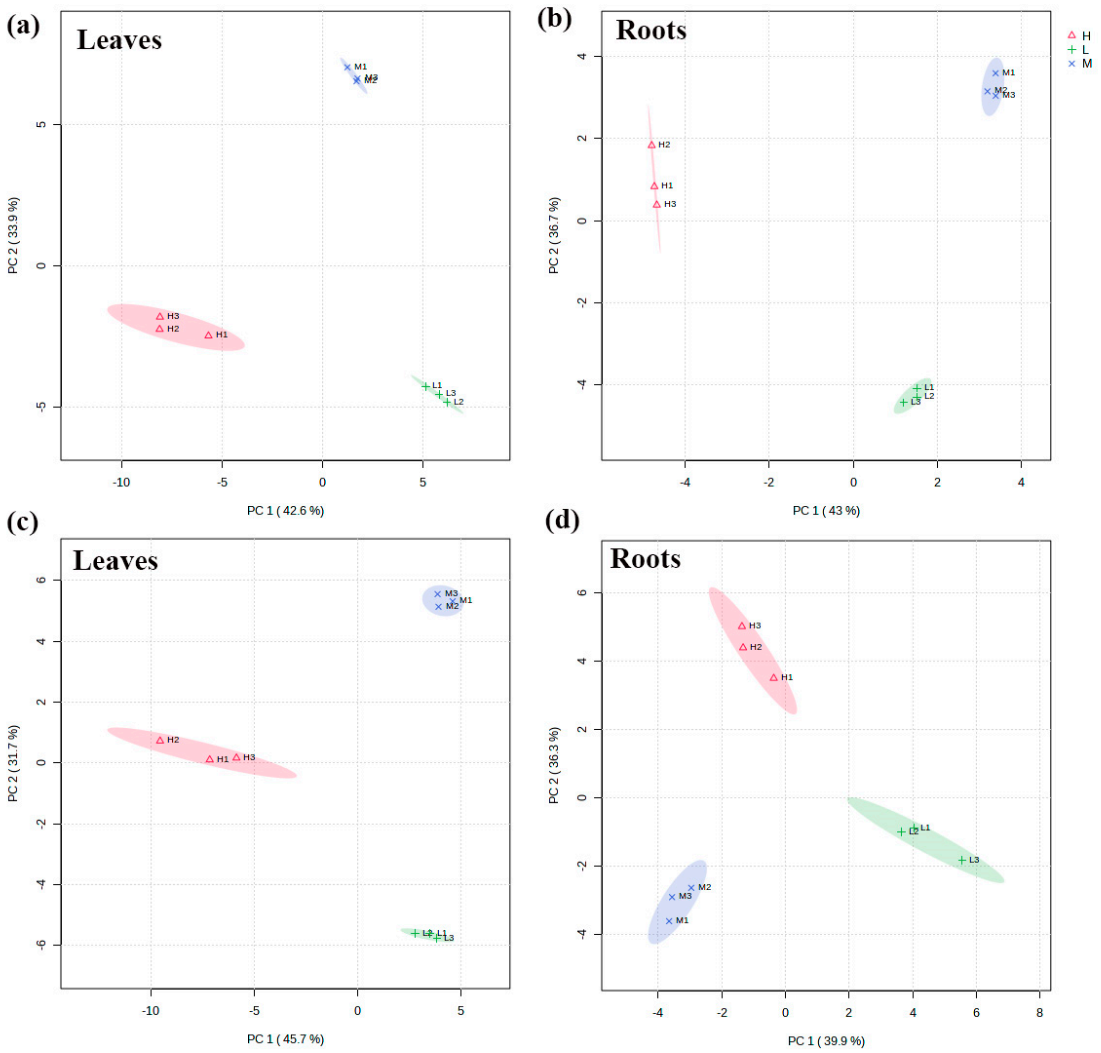

Figure S1 Multivariate analysis of primary metabolites (a, c) and phenolic metabolites (b, d) at different altitudes for leaves and roots.

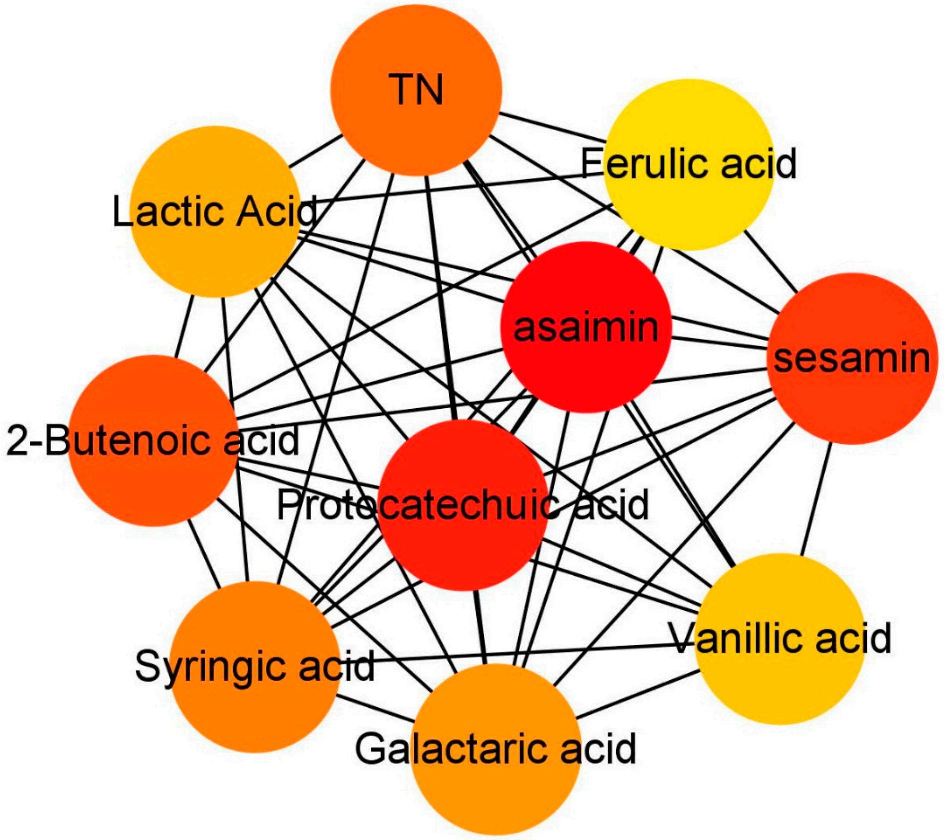

Figure S2 Top 10 nodes in *Asarum* ranked by MCC.
